# Dihydrochalcones in Sweet Tea: Biosynthesis, Distribution and Neuroprotection Function

**DOI:** 10.3390/molecules27248794

**Published:** 2022-12-12

**Authors:** Yong-Kang Wang, Si-Yi Hu, Feng-Yi Xiao, Zhan-Bo Dong, Jian-Hui Ye, Xin-Qiang Zheng, Yue-Rong Liang, Jian-Liang Lu

**Affiliations:** 1 Tea Research Institute, Zhejiang University, Hangzhou 310058, China; 2 Agricultural and Rural Bureau of Pingyang County, Wenzhou 325499, China

**Keywords:** *Lithocarpus litseifolius*, trilobatin, phloridzin, phloretin, metabolic pathway, bioactivity

## Abstract

Sweet tea is a popular herbal drink in southwest China, and it is usually made from the shoots and tender leaves of *Lithocarpus litseifolius.* The sweet taste is mainly attributed to its high concentration of dihydrochalcones. The distribution and biosynthesis of dihydrochaldones in sweet tea, as well as neuroprotective effects in vitro and in vivo tests, are reviewed in this paper. Dihydrochalones are mainly composed of phloretin and its glycosides, namely, trilobatin and phloridzin, and enriched in tender leaves with significant geographical specificity. Biosynthesis of the dihydrochalones follows part of the phenylpropanoid and a branch of flavonoid metabolic pathways and is regulated by expression of the genes, including *phenylalanine ammonia-lyase, 4-coumarate: coenzyme A ligase, trans-cinnamic acid-4-hydroxylase* and *hydroxycinnamoyl-CoA double bond reductase*. The dihydrochalones have been proven to exert a significant neuroprotective effect through their regulation against Aβ deposition, tau protein hyperphosphorylation, oxidative stress, inflammation and apoptosis.

## 1. Introduction

Tea is one of the most popular beverages worldwide. In addition to the traditional tea made from the leaves of *Camellia sinensis* (L.) O. Kunzte, herbal teas are regionally consumed and usually made from the leaves or other tissues of multifarious plants, such as sweet tea (*Lithocarpus litseifolius*), camomile (*Chrysanthemum lavandulifolium*), jasmine (*Jasminum sambac*), *Dracocephalum rupestre*, honeysuckle (*Lonicera japonica*) and *Litsea coreana* var. *lanuginosa*. The *Lithocarpus litseifolius* has usually been called sweet tea or sweet leaf tree by the local residents since the products made from its leaves taste quite sweet. In China, the plucked shoots and tender leaves are often manufactured according to protocols similar to green tea or black tea, the annual output of the sweet tea product is estimated to exceed 2000 t, and these products are commonly consumed by local people in some provinces along the Yangtze River, such as Sichuan, Chongqing, Hunan and Jiangxi. The sweet tea has attracted much more attention recently because of its unique taste and health benefits, which are attributed to dihydrochalcones (DHCs) such as trilobatin, phloridzin and phloretin. DHCs are a class of flavonoids characterized by a basic C_6_-C_3_-C_6_ backbone structure and the absence of a heterocyclic C ring. DHCs are considered to be the primary precursors and represent important intermediates in the synthesis of flavonoids [1,2]. DHCs, especially phloretin, phloridzin and trilobatin, have a variety of health effects, like antioxidant activity [3], anti-inflammation [4,5], antidiabetic activity [6,7], cardioprotection [8], intestinal protection [9], hepatoprotective effect [10], anticancer activity [11] and neuroprotection [12]. With an increase in the population aging, the incidence of neurodegenerative diseases has increased dramatically [13]. Neurodegenerative diseases mainly include Alzheimer’s disease (AD), Parkinson’s disease (PD), stroke and so on. In folk experience, drinking sweet tea can often be used to prevent and treat neurodegenerative diseases, which might be attributed to the effect of DHCs. Nowadays, DHCs have been well studied in *Malus pumila* Mill., being 25.6–113.7 mg/g DW in leaves [14] and 99.9 mg/g DW in immature fruits. These are natural sources for the DHCs’ separation and utilization. However, the sweet tea can also be considered as an alternative source of the DHCs because of its high level of these compounds. This review introduces the physiological and biochemical characteristics of the sweet tea, summarizes the DHCs biosynthesis and the influencing factors, and finally elaborates on the neuroprotection of the sweet tea and its related mechanisms.

## 2. Physio-Biochemical Characteristics of the Sweet Tea

### 2.1. Morphological Characteristics

**Figure 1:**
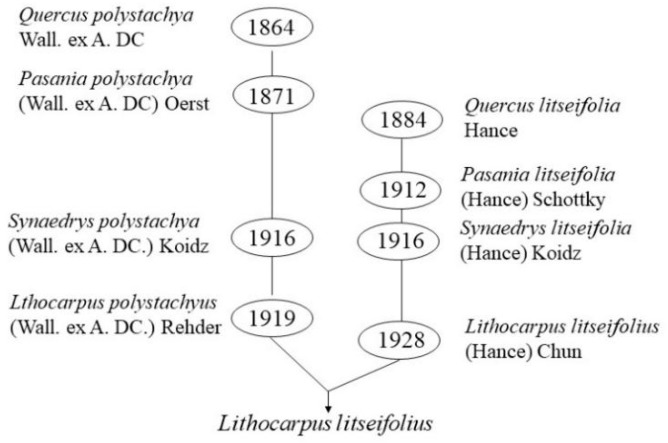
The change of scientific name of the sweet tea.

*Lithocarpus polystachyus* or *Lithocarpus litseifolius* have been used to refer to the sweet tea tree in published reports. According to WFO (www.worldfloraonline.org (accessed on 1 January 2020)), *Lithocarpus polystachyus* is an evergreen arbor in the genus *Lithocarpus* of the family *Fagaceae*. It was first reported as *Quercus polystachya* Wall. ex A. DC. in 1864, then renamed as *Pasania polystachya* (Wall. ex A. DC.) Oerst. in 1871 and *Synaedrys polystachya* (Wall. ex A. DC.) Koidz. in 1916. Later, it was finally designated as *Lithocarpus polystachyus* (Wall. ex A. DC.) Rehder in 1919. *Lithocarpus litseifolius* is also a species belonging to the genus *Lithocarpus* in the family *Fagaceae*. This species was first reported as *Quercus litseifolia* Hance in 1884, then renamed as *Pasania litseifolia* (Hance) Schottky in 1912 and *Synaedrys litseifolia* (Hance) Koidz. in 1916, and finally designated as *Lithocarpus litseifolius* (Hance) Chun in 1928. However, according to FOC (www.eFloras.org, (accessed on 2 March 2021)), these two species are synonyms and belong to the same variety of *Lithocarpus litseifolius*, namely, *Lithocarpus litseifolius* var. *Litseifolius,* as shown in Figure 1, because of their similar morphological characteristics and overlapping geographical distributions. This variety mainly distributes in south China, Laos, northeast Myanmar, northern Vietnam and India [15,16]. In addition to the var. *litseifolius*, another var. *pubescens* Huang has also been found and identified in dense forests of Tian’e County, Guangxi, China. Main morphological differences have been screened out from these two varieties, i.e., the branchlets and infructescences rachis, the cupule size and the fruit ripening time. Glabrous branchlets, sparsely pubescent infructescences rachis, 0.8–1.4 cm cupule diameter and fruit ripening in Jun–Oct has been observed in the former, while puberulent branchlets and infructescences rachis, relatively big cupule (1.2–1.5 cm in diameter) and early fruit ripening time (in April–May) have been witnessed in the latter. In this review, *Lithocarpus litseifolius* is used to refer to all the sweet tea, including the two varieties and the synonymous plants. The sweet tea is a common light-loving and drought-tolerant tree species in the mountain area and usually possesses big elliptic leaves around 8–18 cm in length and 3–8 cm in broadness, ~25 cm male inflorescences in a panicle and ~35 cm female inflorescences with two to six spikes. Its tender leaves or shoots can be picked two to three times a year, but leaf-picking is quite difficult because the tree can grow up to 20 m under natural conditions; therefore, pruning usually has been performed to control the height of the tree below 1.5 m for improving the picking efficiency. Up to now, products in the market are mainly harvested from wild resources since this plant has not been popularly cultivated. Although sweet tea can be propagated through cuttings and seeds similar to the *Camellia sinensis*, sexual propagation through seeds is mainly adopted, which will lead to production difficulties because of the mixed genetic background of the trees.

### 2.2. Concentration of the DHCs and Influence Factors

The sweet tea contains diverse secondary metabolites. Eighteen terpenoids have been identified from cupules [17,18] and 35 flavonoids, including flavones, flavonols, dihydroflavones, isoflavones and DHCs, are detected in stems [19]. Meanwhile, seven triterpenoids are isolated from the leaves and twigs [20], 268 volatiles are identified from young leaves [21] and 68 phenolic compounds have been qualified and quantified from leaves [22]. In this respect, the leaves are the main economic parts of the sweet tea. Among these metabolites, DHCs are the most abundant component in sweet tea. Research showed that the content and composition of the DHCs are influenced by many factors, such as geographical distribution of the trees, leaf maturity and harvest time (Table 1). Yang et al. reported that the abundance of the DHCs is significantly correlated with latitude negatively as well as temperature and soil organic matter and nitrogen content positively [23]. With the decline of latitude, the growth and development of the sweet tea tree will be positively stimulated by an increase in the average annual temperature and rainfall; therefore, increased leaf area, broadened vein distance and accelerated growth rate of the trees have usually been observed in the low latitude area [24,25,26,27,28]. In the sweet tea, trilobatin, phloridzin and phloretin are the main DHCs. The content of these compounds varies with the tissues and organs of the sweet tea tree. With an increase in the maturity of leaves, the phloridzin level increases, and trilobatin decreases [26]; meanwhile, the level of phloretin is relatively low and changes little. Thus, a high level of trilobatin is usually observed in the tender leaves, with a content of about 14–28%, while accumulated phloridzin is witnessed in the mature and old leaves [25]. For the same plant, the content of the three DHCs in tender leaves fluctuates with the harvest time. The content of trilobatin peaks in April, and the phloridzin peaks in April and August [24]. This implies that the DHCs may possess important biological functions for the growth and development of the plant. When the phloridzin biosynthesis was blocked through transgenic operation, the genetically modified ‘Royal Gala’ apple showed a series of severe phenotypic changes, including stunted growth, reduced internode length, narrowed leaf, increased lateral branches and weakened adventitious roots [29]. Exogenous supplementation of phloridzin to the genetically modified apple tree would enhance axial leaf growth and partially restore the leaf to a ‘normal’ shape [30]. Moreover, phloridzin biosynthesis would promote photosynthetic carbon accumulation but limit nitrogen accumulation via the shoot-dependent nitrogen assimilation pathway in apples [31]. In general, high-level phloridzin in mature leaves may help to maintain the morphological and physiological functions of the leaves by regulating photosynthesis and resistance. However, the exact physiological effect has not been clearly revealed till now, and much more research needs to be carried out to elucidate the accumulation mechanism and physiological roles of the DHCs, especially in sweet tea trees. The processing method will remarkably affect the composition and content of the DHCs in the sweet tea. At present, the harvested leaves of the sweet tea have usually been made into “green tea” and “black tea” by adopting traditional tea processing technologies. Fixation, as a characteristic step of green tea processing, can denature the enzymes of fresh leaves and maximally maintain the color and composition of the raw materials through heating at high temperatures in a short time. Four fixation styles, including roller-fixation, microwave-fixation, steam-fixation and fry-fixation, were tested, and the results showed that retention of the different DHCs changed with the fixation styles. The content of the phloretin in products followed the order: fry-fixation > roller-fixation > microwave-fixation> steam-fixation. The level of the phloridzin was highest in products through microwave-fixation, a medium through steam-fixation and roller-fixation, and lowest through fry-fixation. The highest trilobatin was observed in roller-fixation treated products, medium in microwave-fixation and fry-fixation treated products and lowest in steam-fixation treated products [32]. This indicated that the different DHCs might possess various thermal sensitivities and could transform among them during heat treatment. Fermentation is the key step of black tea processing. After fermentation, the contents of total polyphenols, trilobatin and phloridzin in the sweet tea were decreased by 26.36%,10.24% and 39.37%, respectively [33]. This suggested that the redox activity of meta hydroxyls is higher than that of para hydroxyls. In addition, the level of phloridzin extracted from the sweet tea leaves fermented with *Saussurea* bacteria is much higher than that of the naturally fermented and unfermented leaves [34]. Studies also showed that the drying method would impact the level of phenolics in sweet tea, and freeze-drying could retain the highest level of phenolics [35].

## 3. DHCs Biosynthesis and Its Regulation

**Figure 2:**
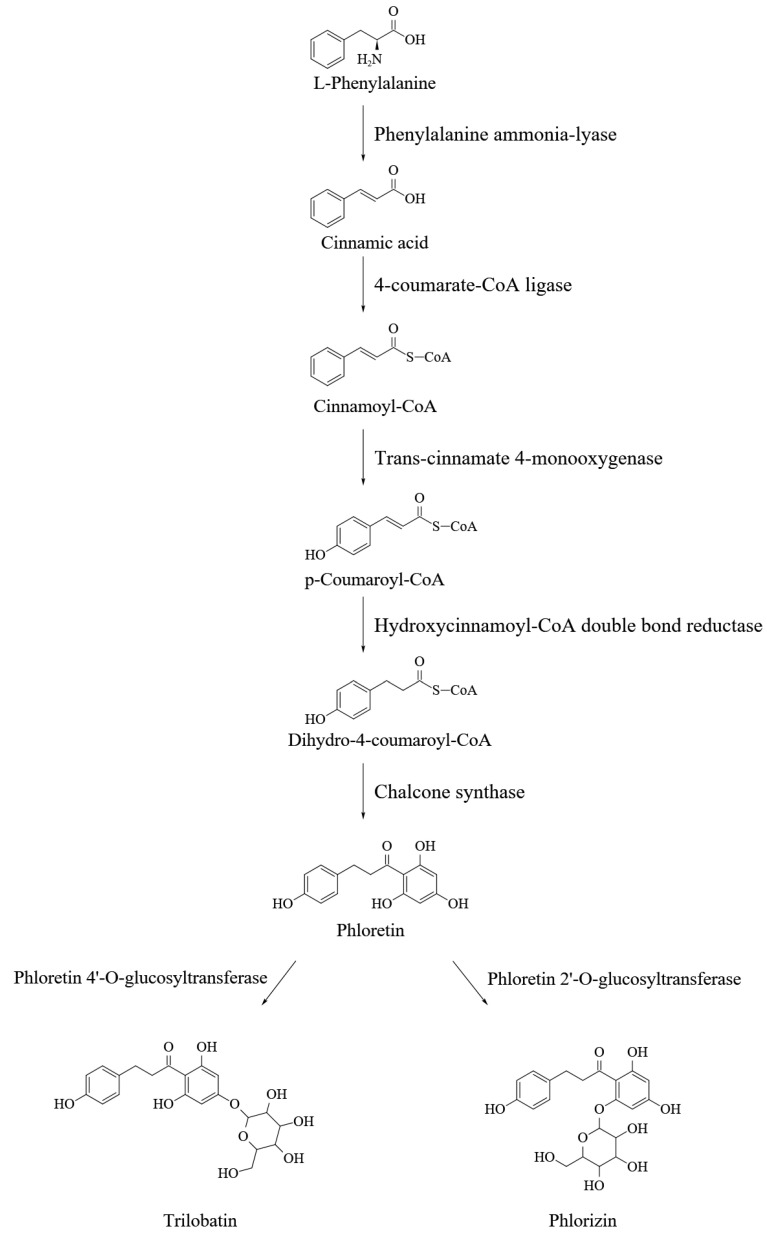
Biosynthesis pathway of the DHCs.

Trilobatin and phloridzin, being positional isomers of the glycosidically bound phloretin, are produced through a side branch of the phenylpropanoids and flavonoids pathways (Figure 2) [38] which is initiated from cleavage of phenylalanine catalyzed by phenylalanine ammonia-lyase (PAL). The first committed step of the DHCs biosynthesis is the conversion of p-dihydrocoumaryl-CoA from p-coumaryl-CoA, which can be catalyzed by a hydroxycinnamoyl-CoA double bond reductase (HCDBR) [39]. Then phloretin will be produced from the p-dihydrocoumaryl-CoA and three units of malonyl-CoA through decarboxylative condensation and cyclization mediated by chalcone synthase (CHS) [40]. Finally, biosynthesis of phloridzin and trilobatin requires the action of UDP-glycosyltransferases (UGTs), also called phloretin-2′-O-glycosyltransferase (P2′GT) and phloretin-4′-O-glycosyltransferase (P4′GT), to attach a glucose moiety at either 2′ or 4′ positions of the phloretin A-ring [1,41]. UGTs catalyze glycosylation of the flavonoids in the plant, and members of the *UGT88Fs* subfamily encode P2′GT and P4′GT, which are responsible for the glycosylation of phloretin in apples [41,42,43,44,45]. *UGT88F1* was the firstly cloned gene encoding the P2′GT in apples [42]. The exogenously expressed P2′GT by *UGT88F1* can specifically glycosylate the phloretin in the presence of UDP-glucose, UDP-xylose and UDP-galactose, but not toward caffeic acid, chlorogenic acid, coumaric acid, cyanidin, 3,4-dihydroxyhydrocinnamic acid, 3-hydroxybenzoic acid, naringenin, 3,4-dihydroxybenzoic acid, catechin, epicatechin, quercetin and rutin. Reports showed that the enzymes encoded by *UGT71A15* and *UGT71K1* of apple can also convert phloretin into phloridzin in vitro [45], while *UGT75L17* of apple encodes P4′GT which catalyzes the 4′ position glycosylation of phloretin to produce the trilobatin in the presence of UDP-glucose [41]. Overexpression of the *UGT75L17* in *Escherichia coli* can be used for efficiently producing trilobatin from phloretin [46]. *UGT71A16*, *UGT71K2* and *UGT88F2* isolated from pear, the relative homolog of *UGT71A15*, *UGT71K1* and *UGT88F1* in apple, can encode the enzymes to synthesize phloridzin from phloretin [45]. This indicated that the biosynthesis limitation of phloridzin and analogs in pear is due to unable formation of phloretin or its precursor(s) rather than a lack of glycosyltransferases. Studies also revealed that trilobatin can be produced through the hydrogenation of the naringin and then hydrolysis with α-L-rhamnosidase in aqueous medium [47]; however, this pathway might not exist under physiological conditions. In the sweet tea tree, *chalcone isomerase* (*CHI*)*, leucoanthocyanidin reductase* (*LAR*)*, flavone 3-hydroxylase* (*F3H*) and *4-coumarate: coenzyme A ligase* (*4CL*) have been cloned, and expression of the *LAR* and *4CL* is positively correlated with DHCs content significantly [48,49,50,51].

The biosynthesis of phloridzin is mainly influenced by light quality, light intensity and photoperiod. The phloridzin content in the sweet tea leaves decreases in the red or blue light treatment and increases in the green light treatment compared with natural light. The content increases with prolonging the illumination time from 8 h to 14 h and with increasing the intensity from 12.5 to 37.5 µmol·m^−2^·s^−1^ in the white light treatment. The change in the phloridzin level is consistent with the expression of *PAL* and *4CL* genes [15]. Thus, the effect of the light on the phloridzin accumulation is mainly regulated by the expression of the genes involved in the early steps of the phenylpropanoid pathway, which might be used to explain the fluctuation of the DHCs along with the change of the season and latitude. In addition, DHCs biosynthesis may also be modulated by hormone level and development status because the accumulation of phloridzin and trilobatin regularly changes with the increase of leaf maturity.

## 4. Neuroprotective Effects of the DHCs

AD, PD and stroke are the main neurological disorders. The pathogenesis of AD is related to amyloid β-protein (Aβ) deposition, formation of nerve fiber tangles (NFTs) through abnormal phosphorylation of tubulin-associated unit (Tau) protein, and synaptic dysfunction. PD syndrome is characterized by the degenerative death of dopaminergic neurons in the midbrain substantia nigra, decreased dopamine concentration in the striatum, and the formation of a Lewy body. The DHCs might exert their neuroprotective effects either directly by inhibiting or alleviating the neurological damage or indirectly by preventing the nervous tissue from oxidative stress, inflammation and apoptosis (Table 2).

### 4.1. Prevention and Treatment of Neurological Diseases

Under normal physiological conditions, amyloid precursor protein(APP) can be digested by α-secretase to produce soluble α-APP fragment (sAPPα) and 83-amino-acid membrane-bound C-terminal fragment (C83), and then the C83 is cleaved by γ-secretase to produce non-toxic fragments. However, when the APP is cleaved by β-secretase, soluble β-APP (sAPPβ) and 99-amino-acid C-terminal (C99) fragments will be generated, and then the C99 will be digested by γ-secretase to produce a variety of Aβ peptides containing 39–42 amino acid residues. The Aβ monomers can spontaneously aggregate and deposit into oligomers, fibrils and senile plaques, which then induce oxidative injury, microglial and astrocytic activity as well as alter kinase/phosphatase activity, eventually leading to neuronal death and AD [62]. Recent reports showed that trilobatin and phloretin can decrease Aβ deposition by down-regulating the expression of *beta-site APP cleaving enzyme 1* (*BACE1*) and consequentially decreasing the β-secretase [53]. Oral administration with 20 mg/kg trilobatin could protect 3 ×FAD (familial Alzheimer’s disease) mice from neurological damage by alleviating Aβ deposition, synaptic degeneration, astrocytosis and microgliosis activation, neuronal loss and cognitive deficits [53]. Phloretin could also suppress Aβ aggregation in the dentate gyrus and CA1 (Cornu Ammonis region 1) regions of the hippocampus in the Aβ-induced AD rats [57]. Furthermore, the Aβ_1–42_ impaired plasticity at the neuronal and presynaptic levels could be restored to normal value by phloretin treatment [56].

**Figure 3:**
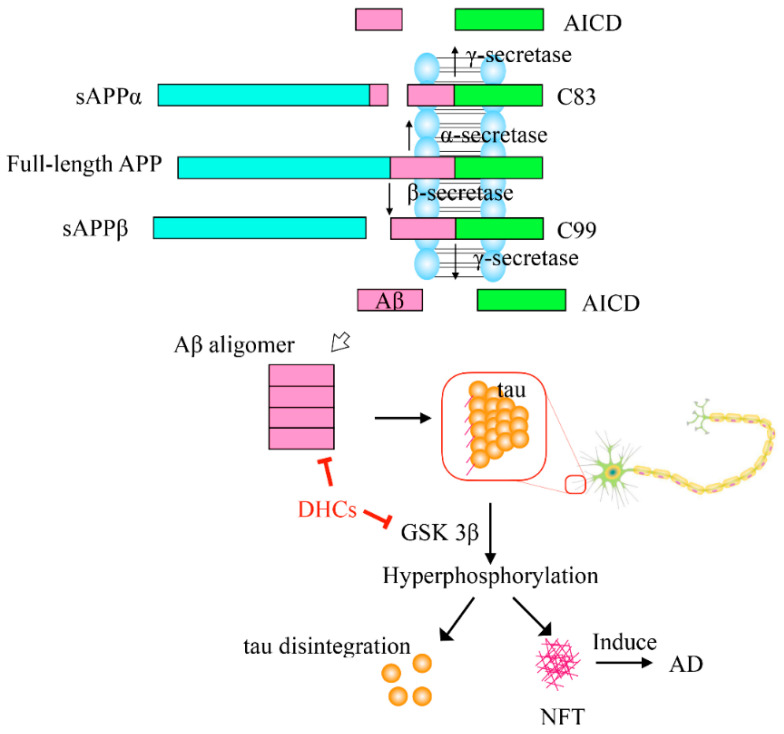
Directly neuroprotective effects of the DHCs. APP, amyloid precursor protein; sAPPα, soluble α-APP fragment; C83, 83-amino-acid membrane-bound C-terminal fragment; AICD, APP intracellular domain; sAPPβ, soluble β-APP; C99, 99-amino-acid C-terminal; Aβ, amyloid β-protein; GSK 3β, glycogen synthase kinase 3β; NFT, Nerve Fiver Tangles. Arrow (→) indicates activation, and the blunt arrow (--**|**) indicates inhibition.

Tau is a main microtubule-associated protein of neurons and plays a critical role in microtubule assembly and stability maintenance. Abnormal hyperphosphorylation of tau neutralizes the basic inhibitory domains and enables tau–tau interaction, resulting in the formation of NFTs in nerve cells, consequently causing the disintegration of tubulins and collapse of the delivery system, eventually leading to the death of the nerve cell. At present, it is well known that glycogen synthase kinase-3β (GSK-3β) and protein phosphatase 2A (PP2A) are the two most important enzymes which can regulate the phosphorylation of tau protein. In mice, treatment of trilobatin can directly inhibit the hyperphosphorylation of tau at Ser396 and Ser202 sites and indirectly alleviate the phosphorylation of tau through suppressing the hyperphosphorylation of GSK-3β which acts as a Tau kinase [53], as shown in Figure 3.

### 4.2. Antioxidative Effect

**Figure 4:**
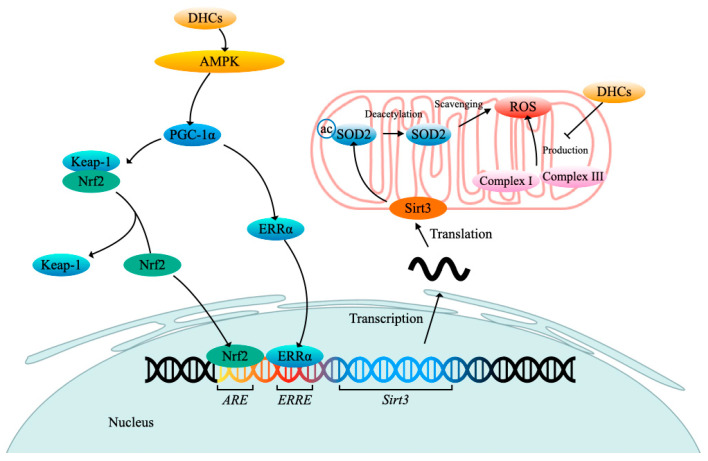
DHCs exert neuroprotection through their antioxidative effect. DHCs, dihydrochalcones; AMPK, AMP-activated protein kinase; PGC-1α, peroxisome proliferators activated receptor-γ coactivator-1α; Keap-1, Kelch-like ECH-associated protein 1; Nrf2, Nuclear respiratory factor 2; ARE, antioxidant response element; ERRα, estrogen-related receptor α; ERRE, ERRα response element; Sirt3, sirtuin 3; SOD2, superoxide dismutase 2; ac, acetylation; ROS, radical oxygen species. Arrow (→) indicates activation, and the blunt arrow (--**|**) indicates inhibition.

It is widely presumed that excessive radical oxygen species (ROS) would lead to a variety of neurological diseases. Trilobatin is able to maintain mitochondrial ROS homeostasis by inhibiting overproduction and promoting the elimination of ROS. Regulation of trilobatin against ROS might carry out mainly by influencing the oxidative stress signal transduction (Figure 4). Pretreatment with trilobatin can stimulate AMP-activated protein kinase (AMPK) phosphorylation which will consequently trigger activation of peroxisome proliferators activated receptor-γ coactivator-1α (PGC-1α) and estrogen-related receptor α (ERRα), and then the activated ERRα will bind to the ERRα response element (ERRE) of sirtuin 3 (*Sirt3*) promoter to activate the transcription of *Sirt3* which encodes a major mitochondria NAD^+^-dependent deacetylase, or the activated PGC-1α will accelerate the disassociation of complex Kelch-like ECH-associated protein 1 (Keap-1)/Nuclear respiratory factor 2 (Nrf2) to provoke translocation of the key transcript factor Nrf2 from the cytoplasm into nucleus which can activate the transcription of *Sirt3* through recognizing and binding to antioxidant response element (ARE) of the *Sirt3* promoter. The elevated Sirt3 will promote superoxide dismutase 2 (SOD2) deacetylation, which can boost ROS scavenging. It was confirmed that trilobatin protected HT22 cells against isoflurane-induced neurotoxicity mainly via activating the Nrf/ARE pathway [55], and phloretin could also eliminate the oxidative stress of the cerebral ischemia/reperfusion rats mainly through activating the Nrf2 defense pathway [60]. The study also showed that trilobatin could reverse the cytotoxicity of HT22 cells induced by the Aβ_25–35_ treatment partially by inhibiting the oxidative injury mediated by mitogen-activated protein kinase p38 (p38)/Sirt3 pathway [52]. In addition, trilobatin can exert antioxidant capacity by improving the activities of NADH-ubiquinone oxidoreductase and ATPase and balancing the NAD^+^/NADH ratio [12]. However, the antioxidative effects of phloretin and phloridzin cannot be achieved by increasing the activity of enzymes such as SOD or catalase (CAT). However, they can directly scavenge the superoxide anions generated from both the electron transfer chain reactions of NADH-ubiquinone oxidoreductase /FMN/Fe-S clusters (complex I) and ubiquinone-cytochrome c oxidoreductase/cytochrome b/ Fe-S clusters (complex III) in human SH-SY5Y neuronal-like cells treated with inducer rotenone [63]. 

### 4.3. Anti-Neuroinflammation and Anti-Apoptosis

**Figure 5:**
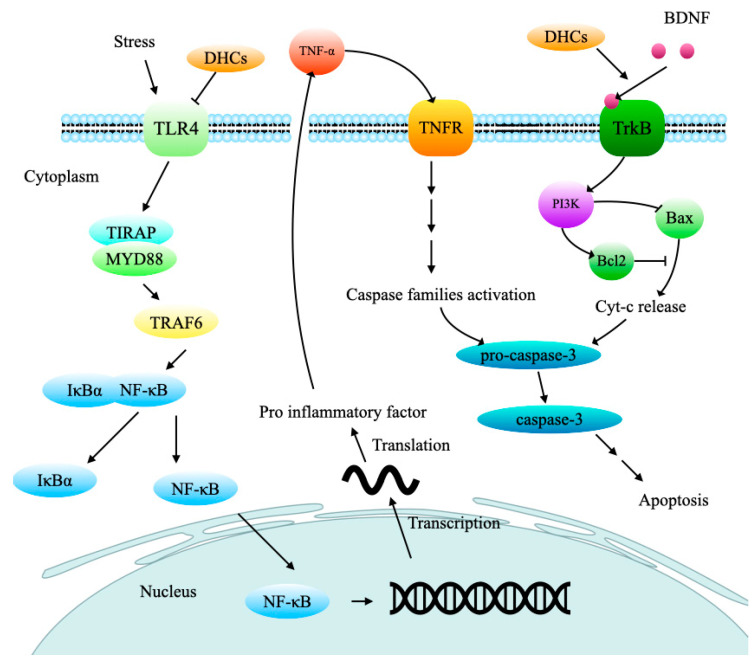
DHCs exert neuroprotective effects through their anti-inflammation and anti-apoptosis. TLR4, Toll-like receptor 4; TIRAP, toll/interleukin 1 receptor domain-containing adaptor protein; MYD88, myeloid differentiation factor 88; TRAF6, TNF receptor-associated factor 6; IκBα, inhibitor of NF-κΒ; NF-κB, nuclear factor κ-B; TNF-α, tumor necrosis factor α; TNFR, tumor necrosis factor receptor; BDNF, brain-derived neurotrophic factor; Trk B, tyrosine kinase receptor B; PI3K, phosphatidylinositol 3- kinase. Arrow (→) indicates activation, and the blunt arrow (--**|**) indicates inhibition.

Neuroinflammation is critical damage resulting in neurological diseases. It has been proved that trilobatin could inhibit the inflammation in middle cerebral artery occlusion (MCAO)-induced cerebral I/R injury rats by suppressing the toll-like receptor 4(TLR4)/ myeloid differentiation factor 88(MyD88)/tumor necrosis factor (TNF) receptor-associated factor 6 (TRAF6) signaling pathway, and down-regulating the phosphorylation level of NF-κBp65 which will restrain the release of proinflammatory factors, including IL-1β, IL-6, TNF-α and inducible nitric oxide synthase (iNOS) [54], as shown in Figure 5. Pretreatment with phloretin could also inhibit the activation of the glial cell and suppress the inflammatory responses [58].

The experiment showed phloridzin could significantly elevate the brain-derived neurotrophic factor (BDNF) level and reduce the acetylcholinesterase (AChE) activity in the hippocampus and cortex of the lipopolysaccharide (LPS)-treated mice [61]. BDNF can bind to tyrosine receptor kinase B (Trk B), which activates phosphatidylinositol 3-kinase (PI3K), and then spurs the anti-apoptosis and protein synthesis [64]. AChE can degrade acetylcholine and terminate the excitatory effect of neurotransmitters on the postsynaptic membrane, and reduced AChE levels can inhibit neuron damage and prevent AD formation. A large amount of AChE was expressed when cells were in the state of apoptosis. The increased AChE protein can not only inhibit cell growth but also enter the nucleus and participate in the formation of apoptotic bodies and promote apoptosis [65]. Another research confirmed that inhibition of AChE activity through pretreatment and treatment with phloretin could improve spatial memory formation during the Morris water maze (MWM) test in scopolamine-induced amnesia mice [59]. Tests also showed that trilobatin could prevent HT22 cells from the injury induced by the Aβ_25–35_ treatment via suppression of the caspase-3-dependent apoptosis pathway. Caspase-3, the most important terminal cleavage enzyme in the process of apoptosis, can be activated by TNF, cyt-c and other caspase families. The activated caspase-3 further enlarges the cascading effects and finally leads to cell death [66].

## 5. Conclusions

The shoots and leaves of *Lithocarpus litseifolius* are rich in DHCs, which taste sweet and possess a variety of physiological effects. Phloretin, trilobatin and phloridzin are the main components of the DHCs. The level of these compounds usually fluctuates along with leaf maturity and distribution location, but some contradictory results have been reported because many samples usually have been collected without uniform picking standards from natural growth trees instead of cultivated plants. The biosynthesis of the DHCs begins with the phenylpropanoid pathway and turns to a branch of the flavonoid pathway at the step from p-coumarinyl CoA to dihydro-4-coumaroyl CoA rather than directly to naringenin chalcone. It has been speculated that the accumulation of the DHCs might be regulated at the transcriptional level at the early steps of the pathway. The DHCs can prevent the neuron tissues from damage by directly inhibiting Aβ deposition and tau hyperphosphorylation as well as indirectly eliminating oxidative stress, inflammation and apoptosis. However, up to now, many issues are not clear, especially why the *Lithocarpus litseifolius* accumulates high levels of DHCs in their leaves, what is the exact biological function of these compounds for the trees and how these compounds are synthesized and metabolized precisely. Meanwhile, many more studies need to be approached for better utilizing the *Lithocarpus litseifolius*, such as collection and improvement of the germplasms, technology development of standardization cultivation, optimization of the processing system and new biological function extension and its mechanism elaboration of the DHCs.

## Figures and Tables

**Table 1:** Effect of the location, picking time and leaf maturity on the content of DHCs in the sweet tea.

Influencing Factors	Content (mg/g)			Reference
	Trilobatin	Phloridzin	Phloretin	
**Picking time**				
April	257.52–279.74	11.44–22.34	0.13–0.55	[24,26]
November	30.94–33.59	143.12–208.31	0.11–0.44	
**Leaf maturity**				
Tender leaf	82.90–279.74	9.30–57.40	1.90–2.50	[24,36,37]
Old leaf	19.30–128.80	144.60–208.31	3.60–4.30	
**Location**				
Sichuan	73.32–278.15	4.89–62.87	0.08–1.25	[24,25,26,33,36,37]
Chongqing	41.87–133.98	14.90–31.69	0.10–1.39	
Guangxi	0.90–198.70	4.80–144.60	4.98	
Hunan	171.95–272.35	8.35–19.25	0.18–1.01	
Jiangxi	69.56–183.84	11.58–49.84	0.27–4.16	
Guizhou	161.42–176.90	5.26–7.46	0.36–0.38	
Yunnan	4.87–264.60	0.61–208.29	0.27–1.55	
Guangdong	0.44–60.62	20.29–57.33	Not mentioned	
Fujian	185.47–244.56	7.83–19.24	0.48–1.19	

**Table 2:** Neuroprotective effects of the DHCs.

Compound and Effects	Treatment	Model	Reference
**Trilobatin**			
Decrease the phosphorylation of p38	12.5, 25, and 50 µM	Aβ_25–35_-induced HT22 cells	[52]
Reduce the production of Aβ by decreasing the BACE1 levels	10 or 20 mg/kg	3 × FAD AD model mice	[53]
Activate the AMPK signaling pathway to respond the oxidative stress	15, 30 and 60 µM	H_2_O_2_-induced injury PC12 cells	[12]
Increase *Sirt3* expression and activity	5, 10 and 20 mg/kg	MCAO-induced focal cerebral ischemia rats	[54]
Activate the Nrf2/ARE pathway	10, 20, and 40 µM	Isoflurane-induced HT22 cells	[55]
**Phloretin**			
Protect synaptophysin and improve neuron cells	5 mg/kg	Aβ1-42-injected male Wistar rats	[56]
Inhibit the Aβ accumulation through antioxidation and anti-inflammation	2.5 and 5 mg/kg	Aβ25–35-induced sporadic Alzheimer’s disease rats	[57]
Inhibit the activation of microglia and astrocytes	5 mg/kg	MPTP-induced Parkinson’s disease mice	[58]
Improve the activity of neuron cells via normalizing the AChE activity and alleviating reactive gliosis	2.5, 5 and 10 mg/kg	Scopolamine induced amnesia mice	[59]
Up-regulate the transcription and translation of *Nrf2*	40 and 80 mg/kg	Cerebral ischemia/reperfusion rats	[60]
**Phloridzin**			
Normalize neural signaling and exhibit anti-inflammatory effect	10 or 20 mg/kg	Lipopolysaccharide-induced cognitive impairment mice	[61]

## Data Availability

Not applicable.
